# Supplementary material for: L-shaped relationship between hemoglobin glycation index and short-term mortality in patients with intracerebral hemorrhage: A retrospective cohort study
Source: PLoS One. 2026 May 8;21(5):e0348436. doi: 10.1371/journal.pone.0348436 (PMC13155603; doi:10.1371/journal.pone.0348436)
Supplement: S1 Table — (DOCX) [file pone.0348436.s001.docx]

**S1 Table. missing number of variables.**

| **Variables** | **Miss.freq** | **Miss.percentage(%)** |
| --- | --- | --- |
| Age | 0 | 0 |
| Gender | 0 | 0 |
| Race | 0 | 0 |
| Heart rate | 1 | < 0.1 |
| SBP | 1 | < 0.1 |
| DBP | 1 | < 0.1 |
| Respiratory rate | 5 | 0.3 |
| SpO2 | 2 | 0.1 |
| SOFA | 1 | < 0.1 |
| GCS | 1 | < 0.1 |
| Congestive heart failure | 0 | 0 |
| Hypertension | 0 | 0 |
| Diabetes | 0 | 0 |
| Chronic Pulmonary Disease | 0 | 0 |
| AKI | 0 | 0 |
| Myocardial Infarction | 0 | 0 |
| Peripheral Vascular Disease | 0 | 0 |
| Sepsis | 0 | 0 |
| WBC | 5 | 0.3 |
| RDW | 6 | 0.4 |
| Platelet | 7 | 0.5 |
| BUN | 0 | 0 |
| Creatinine | 0 | 0 |
| Potassium | 0 | 0 |
| Sodium | 0 | 0 |
| Bicarbonate | 0 | 0 |
| Aniongap | 0 | 0 |
| FBG | 0 | 0 |
| HbA1c | 0 | 0 |
| AST | 383 | 29 |
| ALT | 392 | 29.7 |
| total bilirubin | 440 | 33.3 |
| neutrophils | 964 | 73.1 |
| lymphocytes | 964 | 73.1 |
| PT | 106 | 8.0 |
| INR | 106 | 8.0 |
| Mannitol | 0 | 0 |
| Insulin | 0 | 0 |
| Heparin | 0 | 0 |
| Warfarin | 0 | 0 |
| Beta_blockers | 0 | 0 |
| Diuretic | 0 | 0 |
| Vasoactive drug | 0 | 0 |
| Ventilator | 0 | 0 |
| Cerebral Surgery | 0 | 0 |

Abbreviations: SBP, systolic blood pressure; DBP, diastolic blood pressure; SpO2, oxygen saturation; SOFA, sequential organ failure assessment; GCS, Glasgow coma scale; AKI, acute kidney injury; WBC, white blood cell; RDW, red cell distribution width; BUN, blood urea nitrogen; FBG, fasting blood glucose; HbA1c, Hemoglobin A1c; AST, aspartate aminotransferase; ALT, alanine aminotransferase; PT, prothrombin time; HDL, High Density Lipoprotein; INR, international normalized ratio.
