# Supplementary material for: L-shaped relationship between hemoglobin glycation index and short-term mortality in patients with intracerebral hemorrhage: A retrospective cohort study
Source: PLoS One. 2026 May 8;21(5):e0348436. doi: 10.1371/journal.pone.0348436 (PMC13155603; doi:10.1371/journal.pone.0348436)
Supplement: S2 Table — (DOCX) [file pone.0348436.s002.docx]

**S2 Table. Baseline characteristics between patients with missing HbA1c measurements and included ICH patients**

| **Variables** | **Total**  **(n = 3127)** | **Missing HbA1c**  **(n = 1809)** | **Included**  **(n = 1318)** | **P-value** |
| --- | --- | --- | --- | --- |
| Age (year) | 70(59, 81) | 69 (57, 80) | 72(60, 82) | <0.001 |
| Gender, n (%) |  |  |  | 0.914 |
| Female | 1465 (46.85) | 849 (46.93) | 616 (46.74) |  |
| Male | 1662 (53.15) | 960 (53.07) | 702 (53.26) |  |
| Race, n (%) |  |  |  | <0.001 |
| Non-White | 1154 (36.90) | 623 (34.44) | 531 (40.29) |  |
| White | 1973 (63.10) | 1186 (65.56) | 787 (59.71) |  |
| **Vital signs** |  |  |  |  |
| Heart rate (beats/min) | 81.00 (70.00, 93.00) | 80.00 (70.00, 93.00) | 81.00 (71.00, 92.00) | 0.986 |
| SBP (mmHg) | 137.00 (122.00, 151.00) | 137.00 (121.00, 151.00) | 138.00 (125.00, 151.00) | 0.030 |
| DBP (mmHg) | 74.00 (64.00, 86.00) | 73.00 (63.00, 84.00) | 77.00 (66.00, 88.00) | <0.001 |
| Respiratory rate (times/min) | 18.00 (15.00, 22.00) | 18.00 (15.00, 21.88) | 18.00 (16.00, 22.00) | 0.076 |
| SpO2 (%) | 98.00 (96.00, 100.00) | 98.00 (96.00, 100.00) | 98.00 (96.00, 100.00) | <0.001 |
| **Severity scores** |  |  |  |  |
| SOFA | 3 (1, 4) | 3(1, 5) | 3 (1, 4) | <0.001 |
| GCS | 14 (12, 15) | 14 (11, 15) | 14(12, 15) | 0.123 |
| **Comorbidity,n(%)** |  |  |  |  |
| Hypertension | 2434 (77.84) | 1320 (72.97) | 1114 (84.52) | <0.001 |
| Diabetes | 795 (25.42) | 388 (21.45) | 407 (30.88) | <0.001 |
| Chronic Pulmonary Disease | 399 (12.76) | 255 (14.10) | 144 (10.93) | 0.009 |
| Myocardial Infarction | 279 (8.92) | 158 (8.73) | 121 (9.18) | 0.665 |
| **Treatment** |  |  |  |  |
| Mannitol | 413 (13.21) | 271 (14.98) | 142 (10.77) | <0.001 |
| Heparin | 1847 (59.07) | 971 (53.68) | 876 (66.46) | <0.001 |
| Warfarin | 78 (2.49) | 40 (2.21) | 38 (2.88) | 0.234 |
| Insulin | 2144 (68.56) | 1135 (62.74) | 1009 (76.56) | < 0.001 |
| Beta_blockers | 1224 (39.14) | 652 (36.04) | 572 (43.40) | 0.203 |
| Diuretic | 880 (28.14) | 463 (25.59) | 417 (31.64) | <0.001 |
| Vasoactive drug | 497 (15.89) | 355 (19.62) | 142 (10.77) | <0.001 |
| ventilation | 2040 (65.24) | 1203 (66.50) | 837 (63.51) | 0.082 |
| Cerebral Surgery | 338 (10.81) | 233 (12.88) | 105 (7.97) | <0.001 |
| **Outcomes** |  |  |  |  |
| Hospital mortality, n (%) | 770 (24.62) | 555 (30.68) | 215 (16.31) | <0.001 |
| ICU mortality, n (%) | 471 (15.06) | 326 (18.02) | 145 (11.00) | <0.001 |
| 30-day mortality, n (%) | 858 (27.44) | 580 (32.06) | 278 (21.09) | <0.001 |
| 90-day mortality, n (%) | 1021 (32.65) | 683 (37.76) | 338 (25.64) | <0.001 |

Abbreviations: SBP, systolic blood pressure; DBP, diastolic blood pressure; SpO2, oxygen saturation; SOFA, sequential organ failure assessment; GCS, Glasgow coma scale;
