# Supplementary material for: L-shaped relationship between hemoglobin glycation index and short-term mortality in patients with intracerebral hemorrhage: A retrospective cohort study
Source: PLoS One. 2026 May 8;21(5):e0348436. doi: 10.1371/journal.pone.0348436 (PMC13155603; doi:10.1371/journal.pone.0348436)
Supplement: S3 Table — (DOCX) [file pone.0348436.s003.docx]

**S3 Table. Baseline characteristics between 30-day survival and 30-day mortality group**

| **Variables** | **Total**  **(n = 1318)** | **30-day survival**  **(n = 1040)** | **30-day mortality**  **(n = 278)** | **P-value** |
| --- | --- | --- | --- | --- |
| HGI | -0.23 (-0.63, 0.24) | -0.19 (-0.57, 0.27) | -0.44 (-0.86, 0.08) | <0.001 |
| Age (year) | 72 (60, 82) | 70 (59, 81) | 77 (67, 86) | <0.001 |
| Gender, n (%) |  |  |  | <0.001 |
| Female | 616 (46.74) | 460 (44.23) | 156 (56.12) |  |
| Male | 702 (53.26) | 580 (55.77) | 122 (43.88) |  |
| Race, n (%) |  |  |  | <0.001 |
| Non-White | 531 (40.29) | 397 (38.17) | 134 (48.20) |  |
| White | 787 (59.71) | 643 (61.83) | 144 (51.80) |  |
| **Vital signs** |  |  |  |  |
| Heart rate (beats/min) | 81.00 (70.25, 92.00) | 81.00 (70.75, 92.00) | 81.50 (70.25, 94.00) | 0.516 |
| SBP (mmHg) | 138.00 (125.00, 151.00) | 138.00 (126.00, 150.00) | 137.00 (119.25, 153.00) | 0.385 |
| DBP (mmHg) | 77.00 (66.00, 88.00) | 77.00 (66.00, 88.00) | 74.00 (61.00, 88.00) | 0.074 |
| Respiratory rate (times/min) | 18.00 (16.00, 22.00) | 18.00 (15.38, 22.00) | 19.00 (16.00, 22.00) | 0.047 |
| SpO2 (%) | 98.00 (96.00, 100.00) | 97.00 (96.00, 99.00) | 99.00 (96.00, 100.00) | <0.001 |
| **Severity scores** |  |  |  |  |
| SOFA | 3 (1, 4) | 2(1, 4) | 4 (2., 6) | <0.001 |
| GCS | 14 (12, 15) | 14 (12, 15) | 14(9, 15) | 0.035 |
| **Comorbidity,n(%)** |  |  |  |  |
| Congestive heart failure | 226 (17.15) | 160 (15.38) | 66 (23.74) | <0.001 |
| Hypertension | 1114 (84.52) | 884 (85.00) | 230 (82.73) | 0.353 |
| Diabetes | 407 (30.88) | 315 (30.29) | 92 (33.09) | 0.369 |
| Chronic Pulmonary Disease | 144 (10.93) | 115 (11.06) | 29 (10.43) | 0.766 |
| AKI | 1001 (75.95) | 778 (74.81) | 223 (80.22) | 0.061 |
| Myocardial Infarction | 121 (9.18) | 86 (8.27) | 35 (12.59) | 0.027 |
| Peripheral Vascular Disease | 86 (6.53) | 70 (6.73) | 16 (5.76) | 0.559 |
| Sepsis | 452 (34.29) | 305 (29.33) | 147 (52.88) | <0.001 |
| **Laboratory parameters** |  |  |  |  |
| WBC (K/μL) | 9.90 (7.82, 12.60) | 9.60 (7.60, 12.20) | 11.40 (9.22, 15.17) | <0.001 |
| Hemoglobin(g/dl) | 12.6 (11.2, 13.8) | 12.7 (11.5, 14.0) | 11.8 (10.4, 13.4) | <0.001 |
| Platelet (K/μL) | 209.00 (168.00, 260.75) | 211.00 (172.75, 263.25) | 198.00 (147.00, 254.50) | 0.004 |
| BUN (mg/dL) | 16.00 (12.00, 22.00) | 16.00 (12.00, 21.00) | 19.00 (14.00, 25.00) | <0.001 |
| Creatinine (mg/dL) | 0.90 (0.70, 1.10) | 0.90 (0.70, 1.10) | 1.00 (0.80, 1.20) | 0.007 |
| Potassium (mmol/L) | 4.00 (3.70, 4.30) | 3.90 (3.70, 4.30) | 4.00 (3.70, 4.47) | 0.030 |
| Sodium (mmol/L) | 139.00 (137.00, 142.00) | 139.00 (137.00, 142.00) | 140.00 (137.00, 142.00) | 0.300 |
| Aniongap (mmol/L) | 14.00 (12.00, 16.00) | 14.00 (12.00, 16.00) | 14.00 (12.00, 17.00) | 0.120 |
| PT(s) | 12.50 (11.60, 13.80) | 12.40 (11.60, 13.60) | 13.10 (12.00, 14.80) | <0.001 |
| INR | 1.10 (1.10, 1.30) | 1.10 (1.10, 1.20) | 1.20 (1.10, 1.30) | <0.001 |
| **Treatment** |  |  |  |  |
| Mannitol | 142 (10.77) | 68 (6.54) | 74 (26.62) | <0.001 |
| Heparin | 876 (66.46) | 699 (67.21) | 177 (63.67) | 0.266 |
| Warfarin | 38 (2.88) | 34 (3.27) | 4 (1.44) | 0.105 |
| Insulin | 1009 (76.6) | 770 (74) | 239 (86) | < 0.001 |
| Beta_blockers | 572 (43.40) | 442 (42.50) | 130 (46.76) | 0.203 |
| Diuretic | 417 (31.64) | 302 (29.04) | 115 (41.37) | <0.001 |
| Vasoactive drug | 142 (10.77) | 82 (7.88) | 60 (21.58) | <0.001 |
| ventilation | 837 (63.51) | 619 (59.52) | 218 (78.42) | <0.001 |
| Cerebral Surgery | 105 (7.97) | 68 (6.54) | 37 (13.31) | <0.001 |

Abbreviations: HGI, hemoglobin glycation index; SBP, systolic blood pressure; DBP, diastolic blood pressure; SpO2, oxygen saturation; SOFA, sequential organ failure assessment; GCS, Glasgow coma scale; AKI, acute kidney injury; WBC, white blood cell; RDW, red cell distribution width; BUN, blood urea nitrogen; PT, prothrombin time; HDL, High Density Lipoprotein; INR, international normalized ratio.
