# Supplementary material for: L-shaped relationship between hemoglobin glycation index and short-term mortality in patients with intracerebral hemorrhage: A retrospective cohort study
Source: PLoS One. 2026 May 8;21(5):e0348436. doi: 10.1371/journal.pone.0348436 (PMC13155603; doi:10.1371/journal.pone.0348436)
Supplement: S4 Table — (DOCX) [file pone.0348436.s004.docx]

**S4 Table. Univariate cox proportional analysis for 30-day mortality.**

| **Variables** | **HR(95%CI)** | **P-value** |
| --- | --- | --- |
| Age | 1.03 (1.02 ~ 1.04) | < 0.001 |
| Gender (Male vs Female) | 0.65 (0.51 ~ 0.82) | < 0.001 |
| Race (White vs non-White) | 0.69 (0.55 ~ 0.88) | 0.002 |
| Respiratory rate | 1.03 (1.01 ~ 1.05) | 0.015 |
| SpO2 | 1.06 (1.01 ~ 1.11) | 0.020 |
| SOFA | 1.30 (1.25 ~ 1.35) | < 0.001 |
| GCS | 0.87 (0.84 ~ 0.91) | < 0.001 |
| Congestive Heart Failure(Yes vs No) | 1.56 (1.19 ~ 2.06) | 0.002 |
| Myocardial Infarct (Yes vs No) | 1.46 (1.02 ~ 2.08) | 0.036 |
| Sepsis (Yes vs No) | 2.26 (1.78 ~ 2.86) | < 0.001 |
| WBC | 1.01 (1.01 ~ 1.02) | < 0.001 |
| Hemoglobin | 0.85 (0.81,0.9) | < 0.001 |
| Platelet | 0.99 (0.99 ~ 0.99) | 0.013 |
| BUN | 1.02 (1.01 ~ 1.02) | < 0.001 |
| Creatinine | 1.19 (1.08 ~ 1.30) | < 0.001 |
| Potassium | 1.28 (1.08 ~ 1.52) | 0.004 |
| PT | 1.01 (1.00 ~ 1.03) | 0.114 |
| INR | 1.15 (0.99 ~ 1.34) | 0.061 |
| HGI | 0.79 (0.73 ~ 0.87) | < 0.001 |
| Mannitol (Yes vs No) | 0.56 (0.51,0.61) | < 0.001 |
| Insulin(Yes vs No) | 1.98 (1.41,2.78) | < 0.001 |
| Diuretic (Yes vs No) | 1.93 (1.75,2.14) | < 0.001 |
| Vasoactive drug(Yes vs No) | 0.39 (0.34,0.45) | < 0.001 |
| ventilation(Yes vs No) | 0.51 (0.47,0.56) | < 0.001 |
| Cerebral Surgery(Yes vs No) | 1.36 (1.24,1.48) | < 0.001 |

Abbreviations: SpO2, oxygen saturation; SOFA, sequential organ failure assessment; GCS, Glasgow coma scale; WBC, white blood cell; RDW, red cell distribution width; BUN, blood urea nitrogen; PT, prothrombin time; INR, international normalized ratio, HGI, hemoglobin glycation index.
