# Supplementary material for: L-shaped relationship between hemoglobin glycation index and short-term mortality in patients with intracerebral hemorrhage: A retrospective cohort study
Source: PLoS One. 2026 May 8;21(5):e0348436. doi: 10.1371/journal.pone.0348436 (PMC13155603; doi:10.1371/journal.pone.0348436)
Supplement: Attachment — Submitted filename: Response to Reviewers.docx [file pone.0348436.s006.docx]

Dear Editors and Reviewers,

Thank you for your decision letter and the thoughtful comments on our manuscript entitled “L-shaped relationship between hemoglobin glycation index and short-term mortality in patients with intracerebral hemorrhage: a retrospective cohort study” (Submission ID: PONE-D-25-54592). We sincerely appreciate the time and effort you have invested in reviewing our work. Your constructive feedback has been highly valuable for improving the quality and clarity of our manuscript, and we have carefully addressed all comments in the revised version. We have carefully addressed the comments. For clarity, we have also included point-by-point responses to each comment below this letter.

We would be grateful if you could consider the revised manuscript for publication in *PLOS ONE*.

Thank you once again for your thoughtful comments and for considering our research. We look forward to hearing from you at your earliest convenience.

Best regards,
Yongfeng Ni

Department of Neurosurgery, Anqing First People’s Hospital of Anhui Medical University, Anqing, Anhui, China

E-mail: ahmunyf@126.com

**Here is a point-by-point response to the reviewers’ comments and concerns.**

**Reviewer #1 Comments:**

**Comment 1: Updating References: References [3] and [4] are more than a decade old. I recommend citing the latest epidemiological data and clinical guidelines to emphasize the contemporary significance of this research.**

Response: Thank you for your valuable suggestion on updating the references. We have carefully revised references [3] and [4] with latest epidemiological and clinical studies published in 2022, which better reflect the current evidence and enhance the contemporary significance of this study.The updated references are listed as follows:

3. Goeldlin MB, Mueller A, Siepen BM, Mueller M, Strambo D, Michel P, et al. Etiology, 3-month functional outcome and recurrent events in non-traumatic intracerebral hemorrhage. J Stroke. 2022;24: 266–277. doi:10.5853/jos.2021.01823
4. Ruiz-Sandoval JL, Aceves-Montoya J, Chiquete E, López-Valencia G, Lara-López A, Sánchez-González M, et al. Hospital arrival and functional outcome after intracerebral hemorrhage. Rev Investig Clin Organo Hosp Enfermedades Nutr. 2022;74: 51–60. doi:10.24875/RIC.21000471

**Comment 2: Terminology (Lines 84-86): If the authors wish to emphasize that the utility of HGI in ICH has not yet been "investigated" or "explored," I suggest replacing "elucidated" with one of these more conventional terms. If prior research exists in this specific area, please ensure it is appropriately cited to clarify the unique contribution of this work.**

Response: Thank you for this thoughtful observation. We have carefully reconsidered our terminology and the broader context of existing literature.

We acknowledge that a recent study by Sun et al. (2026) investigated the association between HGI and 30-day mortality in ICH patients using the same MIMIC-IV database, reporting an L-shaped relationship with an inflection point at HGI of 0.78. Their valuable work represents an important contribution to this emerging area of research.

However, we wish to clarify the distinctive contributions of our study that extend and complement the existing evidence:. First, while Sun et al. focused exclusively on 30-day mortality, we examined both 30-day and 90-day mortality. This dual outcome design provides critical insights into intermediate-term prognosis, addressing a gap in understanding how metabolic-related risk persists beyond the acute phase of ICH. Second, our study identified distinct inflection points for 30-day(an HGI of 0.692) and 90-day (an HGI of 0.472) mortality, suggesting that the prognostic threshold may shift over time, which is a novel finding not previously reported. Third, our regression equation for calculating predicted HbA1c ( 0.013×FPG+4.354) differs slightly from that of Sun et al. (0.012×glucose+4.383), reflecting variations in cohort composition and potentially offering complementary perspectives. Fourth, we conducted more extensive stratified analyses, revealing a significant interaction with diabetes status (p for interaction < 0.05). This finding adds granularity to the understanding of HGI’s prognostic utility, clarifying that its predictive value is more pronounced in patients with diabetes and helping target subgroups that may benefit from intensified metabolic monitoring.

Given that Sun et al.’s work was published online on January 15, 2026 (after our initial submission), we were unable to cite it in our original manuscript. We have now updated our Introduction and Discussion sections to appropriately acknowledge this concurrent research while clearly articulating the unique contributions of our study.

**Revisions in the Introduction**: Despite the growing interest in HGI as a prognostic marker, investigation of its utility in forecasting clinical outcomes among patients with ICH has only recently emerged. A concurrent study by Sun et al.[12] demonstrated an association between HGI and 30-day mortality in ICH patients, identifying a threshold effect at an HGI of 0.78. Building upon these foundational findings, our study aims to extend the current understanding by expanding the outcome scope beyond acute-phase mortality, exploring potential time-dependent shifts in prognostic thresholds, and investigating effect modification by clinical factors such as diabetes status. These endeavors may inform more nuanced risk stratification strategies for this clinically vulnerable population. (Manuscript Pages 4-5, Lines 81-90)

**Revisions in the Discussion**: Notably, during the preparation of our manuscript, a concurrent study by Sun et al. [12] reported the first association between HGI and 30-day mortality in ICH patients using the same MIMIC-IV database, identifying a threshold effect at HGI of 0.78. Our study extends these preliminary findings in several important ways. First, we examined both 30-day and 90-day mortality outcomes, revealing that the prognostic threshold exhibits time-dependent variation—the difference between the 30-day inflection point (0.692) and 90-day inflection point (0.472) suggests that the prognostic significance of HGI may evolve dynamically beyond the acute phase of illness. Second, our subgroup analyses demonstrated a significant effect modification by diabetes status (P for interaction < 0.05), indicating that the predictive value of HGI is more pronounced in patients with impaired glucose regulation—a finding that adds a new dimension to the biological interpretation of HGI. Third, although our regression equation for calculating predicted HbA1c (0.013 × FPG + 4.354) differed slightly from that of Sun et al. (0.012 × glucose + 4.383), both studies consistently confirmed the association between low HGI and adverse outcomes, reinforcing the robustness of this metric. Together, these complementary findings suggest that HGI may serve as a valuable tool for metabolic risk assessment in ICH patients, and future research should further elucidate its predictive value across different clinical subgroups and time points. (Manuscript Pages 23-24, Lines 423-322)

Notably, the difference between the 30-day and 90-day thresholds (0.692 vs. 0.472) suggests that the prognostic significance of HGI may evolve over time. This time-dependent threshold effect, which has not been previously reported, provides a rationale for dynamic monitoring of HGI beyond the initial presentation. (Manuscript Page 23, Lines 423-427)

This finding, which was not reported in the concurrent study by Sun et al. [12], adds granularity to the clinical application of HGI and suggests that diabetes status should be considered when interpreting HGI for risk stratification. (Manuscript Page 24, Lines 441-444)

12. Sun S, Huang X, Fei X, Ye F, Gong K. Hemoglobin glycation index predicts mortality in intracerebral hemorrhage: a nonlinear threshold effect. Neurol Sci. 2026;47(1):166. doi:10.1007/s10072-025-08700-y

**Comment 3: Selection Bias (Lines 112-114): The fact that 1,809 patients were excluded due to missing HbA1c measurements—a number higher than the final analysis cohort of 1,318—should be emphasized as a significant risk for selection bias. There is a high probability that the final cohort is skewed toward a population with higher interest in chronic disease management or those suspected of having glucose metabolism disorders. I strongly recommend providing comparative data on the baseline characteristics (e.g., age, severity) of the excluded versus included patients.**

Response: We sincerely thank you for raising this important concern regarding potential selection bias due to the exclusion of 1,809 patients with missing HbA1c measurements. We agree that this issue warrants careful examination, and we have now conducted a comprehensive comparative analysis of baseline characteristics, clinical interventions, and outcomes between the excluded and included ICH patients using the MIMIC-IV database. The detailed results are presented in **S2 Table**.

Our analysis reveals that the two groups were well balanced in core prognostic factors for ICH, including GCS score, gender, heart rate, history of myocardial infarction, and mechanical ventilation use (all P > 0.05). This indicates that the excluded and included cohorts were comparable in terms of neurological severity and critical illness status, which are the most important confounders for ICH prognosis.

Statistically significant differences were observed in certain variables, such as age, diabetes prevalence, insulin use, SOFA score, mortality, and several treatments. However, these differences are fully attributable to real-world clinical testing practices and do not indicate systematic selection bias. First, older patients and those with known diabetes are more likely to undergo HbA1c measurement as part of routine metabolic assessment, explaining the higher age and diabetes prevalence in the included group. Second, the excluded group had slightly higher SOFA scores, reflecting greater multi-organ dysfunction. In such critically ill patients, clinicians prioritize acute resuscitation and often omit non-urgent tests like HbA1c, leading to missing data. Third, the higher mortality in the excluded group is consistent with their greater baseline severity (higher SOFA scores), rather than selection bias. Forth, treatment differences (e.g., higher mannitol, vasoactive drug, and cerebral surgery use in the excluded group) further reflect their more critical condition, while higher heparin, insulin, and diuretic use in the included group align with their higher prevalence of hypertension and diabetes, following standard treatment guidelines.

The clinically explainable differences ensure that the included cohort remains representative of the ICH population in the MIMIC-IV database. Therefore, the core finding—an L-shaped association between HGI and short-term mortality in ICH patients—remains valid and robust.

We have incorporated these findings and corresponding discussion into the revised manuscript and added **S2 Table** to the supplementary information.

**Revisions in the Methods:** To assess potential selection bias, we conducted a comparative analysis of baseline characteristics, clinical interventions, and outcomes between patients with missing HbA1c measurements (n = 1,809) and those included in the final cohort (n = 1,318). (Manuscript Page 6, Lines 119-122)

**Revisions in the Results:**

**Comparison of baseline characteristics between patients with missing HbA1c measurements and included ICH patients**

S2 Table presents the comparison between patients with missing HbA1c measurements (n = 1,809) and those included in the final cohort (n = 1,318). The two groups were well balanced in core prognostic factors for ICH, including GCS score, gender distribution, heart rate, history of myocardial infarction, and mechanical ventilation use, indicating comparable neurological severity and critical illness status between groups.

Statistically significant differences were observed in age, diabetes prevalence, insulin use, SOFA score, mortality, and several treatments. The included group was older and had a higher prevalence of diabetes. The excluded group had slightly higher SOFA scores and higher mortality. Treatment differences were also noted: the excluded group more frequently received mannitol, vasoactive drugs, and cerebral surgery, while the included group had higher rates of heparin, insulin, and diuretics. (Manuscript Page 13, Lines 215-229)

**Revisions in the Discussion**: In addition to the subgroup findings, we carefully evaluated the potential impact of missing data on our cohort composition. The observed differences between patients with missing HbA1c measurements and the included cohort are consistent with real-world clinical testing practices and do not indicate systematic selection bias. The higher prevalence of diabetes and insulin use in the included group reflects routine HbA1c monitoring in patients with known glucose metabolism disorders, whereas older age in this group aligns with the tendency to perform comprehensive metabolic assessments in elderly patients with higher comorbidity burden. Conversely, the excluded group had slightly higher SOFA scores and more frequent use of mannitol, vasoactive drugs, and cerebral surgery, indicating greater acute illness severity. In such critically ill patients, clinicians prioritize life-saving interventions over non-urgent tests like HbA1c, explaining the missing data. The higher mortality in the excluded group is a logical consequence of their greater baseline severity, consistent with the well-established relationship between multi-organ dysfunction and poor outcomes. Importantly, the two groups were well balanced in core ICH prognostic factors (e.g., GCS score, mechanical ventilation use), supporting that the missing HbA1c data are driven by clinical priorities rather than selection bias. Therefore, the included cohort remains representative of the ICH population in the MIMIC-IV database, and our main findings are robust. (Manuscript Pages 24-25, Lines 448-466)

**S2 Table. Baseline characteristics between patients with missing HbA1c measurements and included ICH patients**

| **Variables** | **Total**  **(n = 3127)** | **Missing HbA1c**  **(n = 1809)** | **Included**  **(n = 1318)** | **P-value** |
| --- | --- | --- | --- | --- |
| Age (year) | 70(59, 81) | 69 (57, 80) | 72(60, 82) | <0.001 |
| Gender, n (%) |  |  |  | 0.914 |
| Female | 1465 (46.85) | 849 (46.93) | 616 (46.74) |  |
| Male | 1662 (53.15) | 960 (53.07) | 702 (53.26) |  |
| Race, n (%) |  |  |  | <0.001 |
| Non-White | 1154 (36.90) | 623 (34.44) | 531 (40.29) |  |
| White | 1973 (63.10) | 1186 (65.56) | 787 (59.71) |  |
| **Vital signs** |  |  |  |  |
| Heart rate (beats/min) | 81.00 (70.00, 93.00) | 80.00 (70.00, 93.00) | 81.00 (71.00, 92.00) | 0.986 |
| SBP (mmHg) | 137.00 (122.00, 151.00) | 137.00 (121.00, 151.00) | 138.00 (125.00, 151.00) | 0.030 |
| DBP (mmHg) | 74.00 (64.00, 86.00) | 73.00 (63.00, 84.00) | 77.00 (66.00, 88.00) | <0.001 |
| Respiratory rate (times/min) | 18.00 (15.00, 22.00) | 18.00 (15.00, 21.88) | 18.00 (16.00, 22.00) | 0.076 |
| SpO2 (%) | 98.00 (96.00, 100.00) | 98.00 (96.00, 100.00) | 98.00 (96.00, 100.00) | <0.001 |
| **Severity scores** |  |  |  |  |
| SOFA | 3 (1, 4) | 3(1, 5) | 3 (1, 4) | <0.001 |
| GCS | 14 (12, 15) | 14 (11, 15) | 14(12, 15) | 0.123 |
| **Comorbidity,n(%)** |  |  |  |  |
| Hypertension | 2434 (77.84) | 1320 (72.97) | 1114 (84.52) | <0.001 |
| Diabetes | 795 (25.42) | 388 (21.45) | 407 (30.88) | <0.001 |
| Chronic Pulmonary Disease | 399 (12.76) | 255 (14.10) | 144 (10.93) | 0.009 |
| Myocardial Infarction | 279 (8.92) | 158 (8.73) | 121 (9.18) | 0.665 |
| **Treatment** |  |  |  |  |
| Mannitol | 413 (13.21) | 271 (14.98) | 142 (10.77) | <0.001 |
| Heparin | 1847 (59.07) | 971 (53.68) | 876 (66.46) | <0.001 |
| Warfarin | 78 (2.49) | 40 (2.21) | 38 (2.88) | 0.234 |
| Insulin | 2144 (68.56) | 1135 (62.74) | 1009 (76.56) | < 0.001 |
| Beta_blockers | 1224 (39.14) | 652 (36.04) | 572 (43.40) | 0.203 |
| Diuretic | 880 (28.14) | 463 (25.59) | 417 (31.64) | <0.001 |
| Vasoactive drug | 497 (15.89) | 355 (19.62) | 142 (10.77) | <0.001 |
| ventilation | 2040 (65.24) | 1203 (66.50) | 837 (63.51) | 0.082 |
| Cerebral Surgery | 338 (10.81) | 233 (12.88) | 105 (7.97) | <0.001 |
| **Outcomes** |  |  |  |  |
| Hospital mortality, n (%) | 770 (24.62) | 555 (30.68) | 215 (16.31) | <0.001 |
| ICU mortality, n (%) | 471 (15.06) | 326 (18.02) | 145 (11.00) | <0.001 |
| 30-day mortality, n (%) | 858 (27.44) | 580 (32.06) | 278 (21.09) | <0.001 |
| 90-day mortality, n (%) | 1021 (32.65) | 683 (37.76) | 338 (25.64) | <0.001 |

**Comment 4: Impact of Stress Hyperglycemia (Lines 233-237): Acute stress in conditions like ICH triggers the release of catecholamines and cortisol, leading to transient hyperglycemia (stress-induced hyperglycemia). Since HGI is calculated as the difference between measured HbA1c and predicted HbA1c based on admission glucose, an acute rise in glucose can inflate the predicted value, thereby artifactually lowering the HGI. It remains unclear whether the higher mortality in the low-HGI group is due to HGI itself or if low HGI is simply a proxy for clinical severity (where more severe cases exhibit stronger stress responses). This needs to be addressed through further statistical adjustment or a more in-depth discussion.**

Response: Thank you for your insightful comment regarding the potential confounding effect of stress hyperglycemia on the association between the HGI and short-term mortality in patients with ICH. We agree that acute physiological stress can lead to transient hyperglycemia, which could artifactually lower the calculated HGI. This raises the valid concern that low HGI may be a marker of clinical severity rather than an independent risk factor.

To address this concern, we have taken the following steps. First, in our fully adjusted Cox regression models (Model 3), we included a comprehensive set of variables reflecting disease severity and acute physiological status. These include the SOFA score, GCS, white blood cell count, vital signs, and the use of life-sustaining treatments such as vasoactive drugs and mechanical ventilation. These adjustments help mitigate the confounding effect of illness severity, including the stress response that drives hyperglycemia. Second, we also conducted subgroup analyses stratified by diabetes status, which showed a significant interaction (p for interaction < 0.05). The inverse association between HGI and mortality was more pronounced in patients with diabetes. This suggests that in a population with underlying chronic glycemic dysregulation, the HGI may capture intrinsic glycation tendencies beyond the acute stress response, whereas in non-diabetics, its value might be more susceptible to acute glucose fluctuations induced by stress. This result also indicates that the prognostic value of HGI is not entirely driven by acute stress, and it can serve as an independent prognostic indicator independent of stress in diabetic patients.

We now provide a more nuanced interpretation of our findings, stating that low HGI could reflect both the acute phase response and intrinsic glycation tendencies. We believe these revisions adequately address your concern and clarify the interpretation of HGI in the context of acute illness. The changes made to the manuscript are detailed below.

**Revisions in the Discussion**: First, acute stress responses in severe ICH can trigger sympathetic and hypothalamic–pituitary–adrenal axis activation, elevating catecholamines and cortisol, which promote transient hyperglycemia[24,25]. This acute glucose elevation triggered by physiological stress may substantially increase the estimated HbA1c levels derived from plasma glucose concentrations, leading to a relative decrease in HGI. Consequently, a low HGI in the acute setting may not solely reflect an individual's intrinsic propensity for protein glycation but could also serve as a surrogate marker for the severity of the physiological stress response. Although our multivariable models were adjusted for several indicators of clinical severity, including SOFA score, GCS, and the use of vasoactive drugs, to mitigate this confounding effect, residual confounding cannot be entirely excluded. Li et al. carried out a multicenter investigation that enrolled 586 individuals diagnosed with spontaneous ICH and determined that stress-induced hyperglycemia closely correlated with adverse early clinical outcomes[26]. Similarly, a retrospective analysis by Zhang et al. including 880 ICH patients showed that even after adjusting for confounders, the stress hyperglycemia ratio remained a stand-alone prognostic indicator of unfavorable outcomes, underscoring its strong prognostic value[27]. These findings imply that acute-phase stress hyperglycemia can distort the assessment of chronic glycemic control, resulting in an artificially low HGI that correlates with worse clinical prognoses among patients with ICH. The more pronounced association observed in patients with diabetes suggests that HGI may capture different risk information depending on the metabolic context. This finding supports the notion that in non-diabetic patients, HGI may be more susceptible to acute stress-related fluctuations, whereas in diabetic patients, it may better reflect intrinsic glycation tendencies with independent prognostic value. Future studies incorporating serial glucose measurements or direct biomarkers of the stress response (e.g., cortisol, catecholamines) would help to further disentangle the independent contribution of HGI from that of acute stress hyperglycemia in ICH prognosis.(Manuscript Pages 21-22 ,Lines 373-400)

**Comment 5: Unadjusted Major Prognostic Factors (Lines 350-353): As acknowledged by the authors, the MIMIC-IV database lacks key ICH-specific prognostic factors such as hematoma volume, location, and expansion. If these factors correlate with HGI, the ability to evaluate HGI's independent predictive power is limited. Please consider whether it is possible to supplement this information for a subset of the sample to perform a sensitivity analysis.**

Response: We would like to sincerely thank you for this insightful comment, which highlights an important limitation of our study. We fully agree that hematoma characteristics are among the strongest predictors of outcome in ICH patients, and the inability to adjust for these factors may confound the observed association between HGI and mortality.

Regarding the request to supplement this information for a subset of the sample, we carefully considered this possibility. Unfortunately, the MIMIC-IV database, as a purely administrative and clinical repository derived from electronic health records, does not contain neuroimaging data or radiologist reports. Accessing and re-analyzing the original imaging data for even a subset of patients would require separate institutional approvals and direct access to the source hospitals' imaging archives, which is beyond the scope of this retrospective database study. Therefore, a sensitivity analysis incorporating these variables is not feasible within the current framework. However, in our fully adjusted models (Model 3), we included the GCS score and SOFA score. GCS is a well-established clinical surrogate for the neurological impact of ICH, which correlates strongly with hematoma volume and location. Adjusting for GCS helps to indirectly account for the clinical severity resulting from these unmeasured radiographic factors.

**Revisions in the Discussion**: Second, important clinical variables such as hematoma volume, location, and expansion—which are known to be strong predictors of ICH outcomes—were not available in the MIMIC database and therefore could not be included in the analysis. This represents a significant limitation, as these radiographic factors are major determinants of neurological deterioration and mortality in ICH patients [31]. If these factors are correlated with HGI, the inability to adjust for them could introduce residual confounding. For example, patients with larger hematomas may experience more profound stress responses, leading to lower HGI values. Such confounding might lead to an overestimation of the independent association between HGI and mortality. To partially mitigate this concern, we included the GCS score in our fully adjusted models. GCS is a well-established clinical surrogate for the neurological impact of ICH and has been shown to correlate strongly with hematoma volume and location[32,33], this adjustment is imperfect. Ideally, future prospective cohort studies should be designed to collect standardized neuroimaging data to rigorously evaluate whether HGI provides incremental prognostic value beyond established ICH-specific radiographic predictors. (Manuscript Page 26, Lines 475-490)

31. Meyrat R, Vivian E, Dulaney B, Barrera Gutierrez JC. Enhanced prediction of in-hospital mortality in intracerebral hemorrhage: impact of serial neurological and radiological reassessment with the ICH Score at 6 hours postadmission. J Neurosurg. 2025;144(2):442-451. doi:10.3171/2025.7.JNS251312

32. Peta B, Mallela SR. Evaluation of Risk Factors and Prognostic Outcomes in Patients with Intracerebral Hemorrhage: A Clinical Study From a Tertiary Care Hospital in South India. Cureus. 2025;17(8): e90871. doi:10.7759/cureus.90871

33. Cindea C, Saceleanu V, Tudor V, et al. Prognostic Factors and Clinical Outcomes of Spontaneous Intracerebral Hemorrhage: Analysis of 601 Consecutive Patients from a Single Center (2017-2023). NeuroSci. 2025;6(3):77. doi:10.3390/neurosci6030077

Thank you very much for your thoughtful comments and suggestions.

**Reviewer #2 Comments:**

**Comment 1: Discussion of Confounding Factors: While the authors acknowledge that hematoma volume, location, and expansion were not available in the MIMIC database, the Discussion would benefit from a more detailed explanation of how the absence of these established ICH prognostic markers might impact the independent strength of the HGI association.**

Response: Thank you very much for your constructive comment. We agree that a more detailed discussion of how the absence of key factors— hematoma volume, location, and expansion—might affect the interpretation of our findings would strengthen the manuscript.

In response, we have substantially expanded the relevant section of the Discussion to explicitly address this limitation and its potential impact. Specifically, we have made the following revisions: First, we now explicitly state that if these hematoma characteristics are correlated with HGI (e.g., patients with larger hematomas may experience more profound stress responses, leading to lower HGI values), the inability to adjust for them could introduce residual confounding. Such confounding might lead to an overestimation of the independent association between HGI and mortality. Second, we explain that although direct radiographic data were unavailable, we incorporated the GCS score in our fully adjusted models. GCS is a well-established clinical surrogate for the neurological impact of ICH and has been shown to correlate strongly with hematoma volume and location. We acknowledge that while this adjustment helps mitigate the confounding effect, it remains an imperfect substitute. Lastly, we explicitly call for prospective cohort studies designed to collect standardized neuroimaging data to rigorously evaluate whether HGI provides incremental prognostic value beyond established ICH-specific radiographic predictors.

**Revisions in the Discussion**: Second, important clinical variables such as hematoma volume, location, and expansion—which are known to be strong predictors of ICH outcomes—were not available in the MIMIC database and therefore could not be included in the analysis. This represents a significant limitation, as these radiographic factors are major determinants of neurological deterioration and mortality in ICH patients[31]. If these factors are correlated with HGI, the inability to adjust for them could introduce residual confounding. For example, patients with larger hematomas may experience more profound stress responses, leading to lower HGI values. Such confounding might lead to an overestimation of the independent association between HGI and mortality. To partially mitigate this concern, we included the GCS score in our fully adjusted models. GCS is a well-established clinical surrogate for the neurological impact of ICH and has been shown to correlate strongly with hematoma volume and location[32,33], this adjustment is imperfect. Ideally, future prospective cohort studies should be designed to collect standardized neuroimaging data to rigorously evaluate whether HGI provides incremental prognostic value beyond established ICH-specific radiographic predictors.(Manuscript Page 26, Lines 475-490)

31. Meyrat R, Vivian E, Dulaney B, Barrera Gutierrez JC. Enhanced prediction of in-hospital mortality in intracerebral hemorrhage: impact of serial neurological and radiological reassessment with the ICH Score at 6 hours postadmission. J Neurosurg. 2025;144(2):442-451. doi:10.3171/2025.7.JNS251312

32. Peta B, Mallela SR. Evaluation of Risk Factors and Prognostic Outcomes in Patients with Intracerebral Hemorrhage: A Clinical Study From a Tertiary Care Hospital in South India. Cureus. 2025;17(8): e90871. doi:10.7759/cureus.90871

33. Cindea C, Saceleanu V, Tudor V, et al. Prognostic Factors and Clinical Outcomes of Spontaneous Intracerebral Hemorrhage: Analysis of 601 Consecutive Patients from a Single Center (2017-2023). NeuroSci. 2025;6(3):77. doi:10.3390/neurosci6030077

**Comment 2: Clarification of HGI Definition: In certain sections, the authors should ensure consistent clarity that HGI is the difference between observed and predicted HbA1c. Explicitly emphasizing that "lower HGI" reflects less non-enzymatic glycation than predicted by ambient glycemia would help the reader interpret the biological implications discussed, such as stress-induced hyperglycemia or anemia.**

Response: We thank you for this valuable suggestion to improve the clarity and consistency of our manuscript. We agree that a precise and consistently communicated definition of HGI is essential for readers to correctly interpret its clinical implications in the context of stress-induced hyperglycemia and anemia. In response to this comment, we have carefully reviewed the manuscript and made the following revisions to ensure consistent clarity throughout.

**Revisions in the Methods:** Thus, a positive HGI indicates that the observed HbA1c is higher than predicted from ambient glucose levels (i.e., more non-enzymatic glycation than expected), whereas a negative HGI indicates that the observed HbA1c is lower than predicted, reflecting less glycation than expected based on the prevailing glucose concentration. (Manuscript Page7, Lines 138-142)

**Revisions in the Discussion:**  Consequently, a low HGI in the acute setting may not solely reflect an individual’s intrinsic propensity for protein glycation but could also serve as a surrogate marker for the severity of the physiological stress response. (Manuscript Page 21, Lines 378-380)

This occurs because the measured HbA1c, the “observed” value in the HGI calculation, is artifactually decreased, while the HbA1c predicted from plasma glucose remains unchanged, leading to a more negative HGI. (Manuscript Page 22, Lines 402-405)

**Comment 3: Typographical Note: On page 8, line 57, there appears to be a minor grammatical omission: "risk. holds potential" should likely be revised to "risk and holds potential".**

Response: Thank you for this careful reading of our manuscript. We agree that this is a typographical error, and we have revised the sentence accordingly.This correction ensures grammatical completeness and improves readability. We appreciate the your attention to detail. **The revised sentence now reads as follows:** An L-shaped association exists between HGI and short-term mortality in ICH patients, with low HGI indicating substantially higher risk and holds potential as a novel prognostic indicator for facilitating early risk stratification in patients with acute ICH.

Thank you very much for your thoughtful insightful comments.

We tried our best to improve the manuscript and make some changes in the revised manuscript. However, these modifications did not alter the content and structure of the paper.

We sincerely appreciate the Editors' and Reviewers' diligent work and hope that the revisions now meet your approval.

Once again, we are grateful for your constructive comments and suggestions.
